# Identification of *Burkholderia thailandensis* with novel genotypes in the soil of central Sierra Leone

**DOI:** 10.1371/journal.pntd.0007402

**Published:** 2019-06-14

**Authors:** Emma Birnie, Senne van ’t Hof, Anne Bijnsdorp, Yembeh Mansaray, Erdi Huizenga, Arie van der Ende, Floor Hugenholtz, Martin P. Grobusch, W. Joost Wiersinga

**Affiliations:** 1 Center for Experimental and Molecular Medicine (CEMM), Amsterdam UMC, location AMC, University of Amsterdam, Amsterdam, the Netherlands; 2 Center of Tropical Medicine and Travel Medicine, Department of Infectious Diseases, Amsterdam UMC, location AMC, University of Amsterdam, Amsterdam, the Netherlands; 3 Lion Heart Medical Centre, Yele, Sierra Leone; 4 Department of Medical Microbiology, Amsterdam UMC, location AMC, Amsterdam, the Netherlands; 5 Department of Infectious Diseases, Amsterdam UMC, location AMC, University of Amsterdam, Amsterdam, the Netherlands; 6 Masanga Medical Research Unit, Masanga, Sierra Leone; University of Texas Medical Branch, UNITED STATES

## Abstract

**Background:**

The soil-dwelling bacillus *Burkholderia pseudomallei* is the etiological-agent of the neglected and life-threatening emerging infection melioidosis. The distribution of *B*. *pseudomallei* in West Africa is unknown. In the present study we aimed to determine whether *B*. *pseudomallei* and *B*. *thailandensis* are present in the environment of central Sierra Leone.

**Methodology/Principal findings:**

In June-July 2017, we conducted an environmental surveillance study–designed in accordance with existing consensus guidelines—in central Sierra Leone. A total of 1,000 soil samples (100 per site) were collected and cultured. *B*. *pseudomallei* was not identified in the soil, but we identified seven novel *B*. *thailandensis* sequence types with multi-locus sequence typing (MLST) and 16S rRNA gene sequence analyses.

**Conclusions/Significance:**

The presence of *B*. *pseudomallei* was not demonstrated, however, multiple novel *B*. *thailandensis* sequence types were identified. More environmental and sequencing studies are needed to further understand the genetic diversity, evolution and virulence of these emerging organisms.

## Author summary

The environmental bacterium *Burkholderia pseudomallei* is the cause of melioidosis, an often-fatal but neglected infection prevalent across tropical areas. *B*. *thailandensis* is a member of the *B*. *pseudomallei* complex, rarely causes disease in humans and is considered a-virulent. Modelling studies have estimated a high prevalence of *B*. *pseudomallei* in western Africa. In this study, we performed an environmental surveillance study in the West African country of Sierra Leone. Remarkably, we could not demonstrate the presence of *B*. *pseudomallei* in the soil of Sierra Leone. However, by both culture and sequencing methods, we identified multiple *B*. *thailandensis* strains and novel genotypes. Patients with debilitating *B*. *thailandensis* infection have been occasionally reported in among others Southeast Asia and the US. Environmental and sequencing studies on both *B*. *pseudomallei* and *B*. *thailandensis* are essential to further understand the genetic diversity and evolution of these neglected but emerging organisms.

## Introduction

The Gram-negative environmental bacterium *Burkholderia pseudomallei* is the etiological agent of melioidosis, an emerging but neglected infectious disease. Disease presentations vary from abscess formation to fulminant sepsis [1]. Melioidosis has a mortality up to 50% in low resource settings and is predominantly found in Southeast-Asia and northern Australia [1]. Infection with *B*. *pseudomallei* primarily occurs in people who are in regular contact with soil and water [1,2]. *B*. *thailandensis* is a member of the *B*. *pseudomallei* complex, is considered a-virulent [3,4] and rarely causes disease in humans [5–10]. Knowledge about the global distribution of *B*. *pseudomallei* and *B*. *thailandensis*, however, is limited.

Patients from sub-Saharan Africa reported with melioidosis are few and isolated (e.g. the The Gambia, Burkina Faso, Nigeria and Gabon), which most probably is the result of under-recognition and under-reporting. These cases may represent the ‘Tip of the Iceberg’ [1,11]. From the West African country of Sierra Leone, only one case of melioidosis has been reported [12]. Modelling studies, however, estimate that in Sierra Leone annually hundreds of patients suffer from melioidosis, of which the vast majority will die [13]. The tropical climate, heavy rains and abundant rice farming in central Sierra Leone all contribute to the high pre-odds likely-hood for the presence of *B*. *pseudomallei* and *B*. *thailandensis* in its soils [13]. In the present study we aim to determine whether *B*. *pseudomallei* and *B*. *thailandensis* are present in the soil of central Sierra Leone.

## Methods

**Fig 1:**
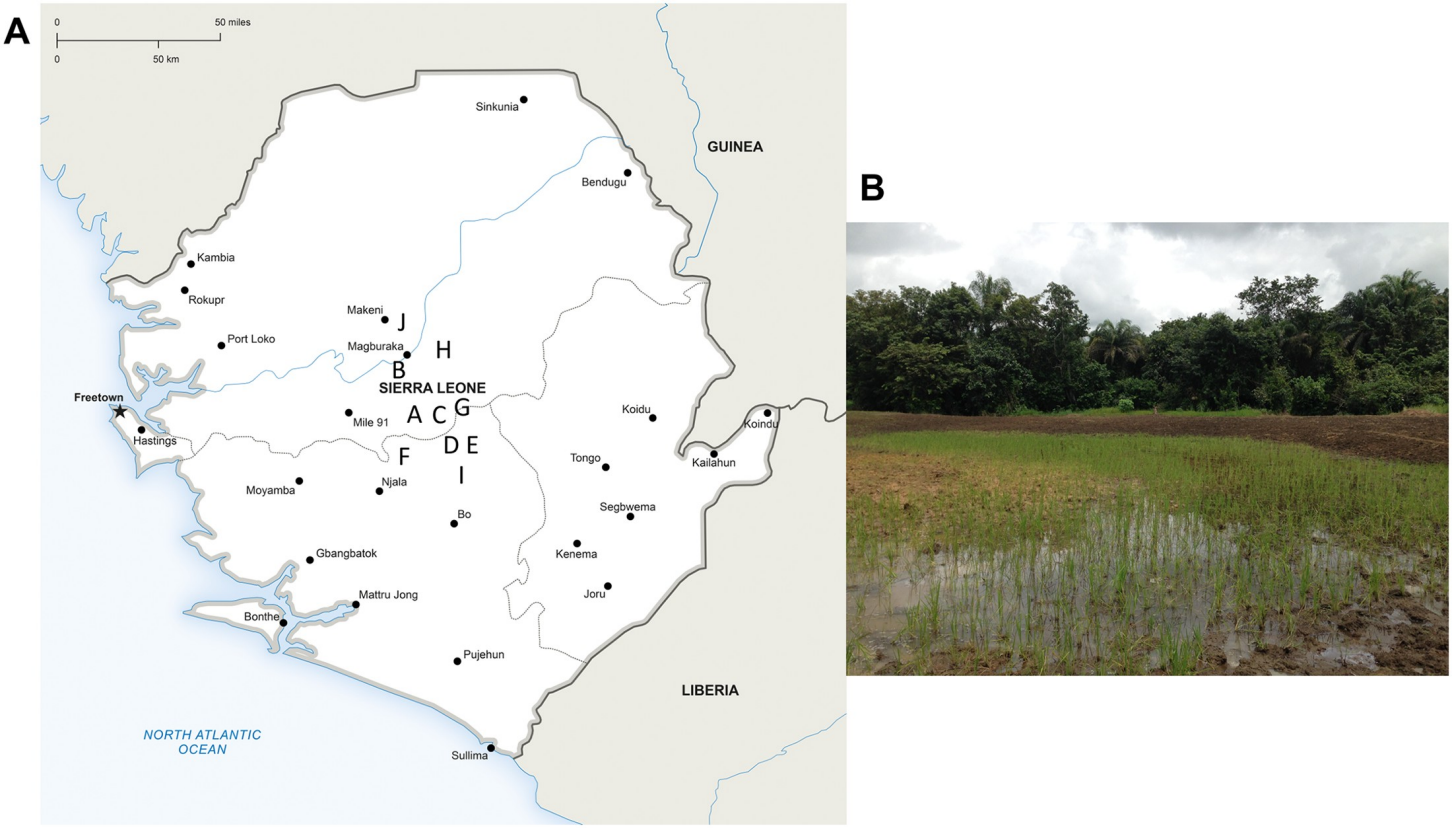
Location of soil sampling sites in Sierra Leone and phylogenetic tree of isolated *B*. *thailandensis* isolates. (A) Sierra Leone, showing the location of 10 soil sampling sites to test for the presence of *B*. *pseudomallei*, June-July 2017. (B) Rice farm Sierra Leone, site E. Image adapted from onestopmap.com.

During the rainy season (June-July 2017), an environmental surveillance study–designed in accordance with existing consensus guidelines [14]—was conducted in Tonkolili and Bombali districts, central Sierra Leone (Fig 1A and 1B). Ten different sites were selected based on local maps and consultations with inhabitants on factors known to be associated with *B*. *pseudomallei* and *B*. *thailandensis* presence (e.g., wet soil such as rice paddies or land use such as goat and cattle farming) [13,14]. Oral informed permission was obtained from landowners and written informed permission from the paramount chief of Yele, Sierra Leone, prior to soil sampling. We used a fixed-interval sampling grid of five meters between soil samples. Thirty grams of soil from a depth of 65 cm [15] was taken for each sample, stored from direct sunlight and kept at room temperature until further processing (for details on geographical features and distribution see Table 1). Culture of suspected *Burkholderia* isolates from soil samples was done as described previously [14,16]. First, 10 grams of each soil sample was diluted in 10 mL of threonine-basal solution containing colistin at 50 mg/L (TBSS-C50 broth) and crystal violet. This mixture was vortexed and subsequently incubated at 40 °C for 48 hours. Ten μL of the upper layer of enrichment medium was subcultured onto an Ashdown-agar plate and checked for suspected *Burkholderia* colonies every 24 hours for a period of 7 days. Initial identification of suspected *Burkholderia* growth was done by colony morphology, positive oxidase test result, antimicrobial drug susceptibility pattern (susceptible to amoxicillin-clavulanic and resistant to gentamicin and colistin) and latex-agglutination tests [14,16] (see S1 Fig for a schematic overview of the culture methodology). All experiments were conducted in a biosafety level 3 laboratory.

**Table 1:** Geographical features and distribution of *Burkholderia* strains at ten sampling sites in the Tonkolili and Bombali District, central Sierra Leone (2017).

Site	Nearest village; location site*	Land use	Soil description	Sample holes positive for *Burkholderia thailandensis*
**A**	Rochain; lat. N 0.8°24’49.83, long. W 11°51’17.82	Rice paddy, next to stream	Clay	22
**B**	Matamp; lat. N 0.8°32’9.658, long. W 11°56’30.63	Rice paddy, wet	Clay, mud	3
**C**	Yele; lat. N 0.8°24’46.17, long. W 11°50’38.28	Groundnut farm, goats, next to hospital	Clay	0
**D**	Mabara; lat. N 0.8°25’54.51, long. W 11°52’50.15	Wet grassland	Clay, mud, brown orange, black, grey	0
**E**	Makonkorie; lat. N 0.8°33’13.99, long. W 11°48’54.27	Rice paddy	Clay, mud	0
**F**	Mayessie Junction; lat. N 0.8°26’52.63, long. W 11°57’05.31	Rice paddy	Clay, mud	0
**G**	Patifu Mayawa; lat. N 0.8°26’23.00, long. W 11°49’32.38	Swamp, goats	Sandy, mud	0
**H**	Matotoka; lat N 0.8°39’20.14, long. W 11°51’23.56	Rice paddy, after harvest groundnut and potato farm	Clay, red soil	0
**I**	Ferry; lat. N 0.8°24’58.66, long. W 11°69’57.46	Rice paddy, wet	Sandy soil	0
**J**	Matene (near Makeni); lat. N 0.8°47’46.56, long. W 12°01’31.74	Rice paddy, dry	Dry, sandy, yellow, light brown	0

*lat. = latitude; long. = longitude.

All isolates were identified as *B*. *thailandensis* by MLST as well as 16S sequence analyses (see S1 Table). *B*. *pseudomallei* was not found. Four *B*. *thailandensis* isolates corresponded to the earlier described sequencing type 73 (ST73) [17], but the remaining 28 isolates were of seven novel sequence types (ST1677 through ST1683). ST1677 and ST1680 are single locus variants of ST73, while ST1678, ST1679, ST1681 and ST1682 are double locus variants of ST73 and ST1683 differed at three loci from ST73 (see appendix).

Multi-locus sequence typing (MLST) and 16S rRNA gene sequence analyses were used to determine the species of the isolated *Burkholderia* isolates using the MiSeq platform (Illumina, San Diego, CA, USA) as described previously [16]. Assembly of the reads was performed with help of SPAdes 3.9. MLST data analysis was performed based on partial sequences of seven housekeeping genes (see appendix). Allele numbers and sequence types deduced from MLST allelic profiles were assigned using the BIGSdb *B*. *pseudomallei* database (https://pubmlst.org/bpseudomallei/). Cluster analysis was subsequently performed in the MEGA 6.06 using the UPGMA algorithm and the Jukes-Cantor model. Bootstrap test was performed for 500 repetitions.

### Ethics statement

Oral informed permission was obtained from landowners and written informed permission from the paramount chief of Yele, Sierra Leone, prior to soil sampling.

## Results/Discussion

A total of 1,000 samples (100 per site) were collected at ten sampling sites in the Tonkolili and Bombali District, central Sierra Leone (see Table 1). Initial identification methods [14,16], led to the isolation of 32 *Burkholderia* strains from 25 soil samples. Four *Burkholderia* strains showed a negative latex-agglutination test; the rest showed (possible) positive latex-agglutination test results.

**Fig 2:**
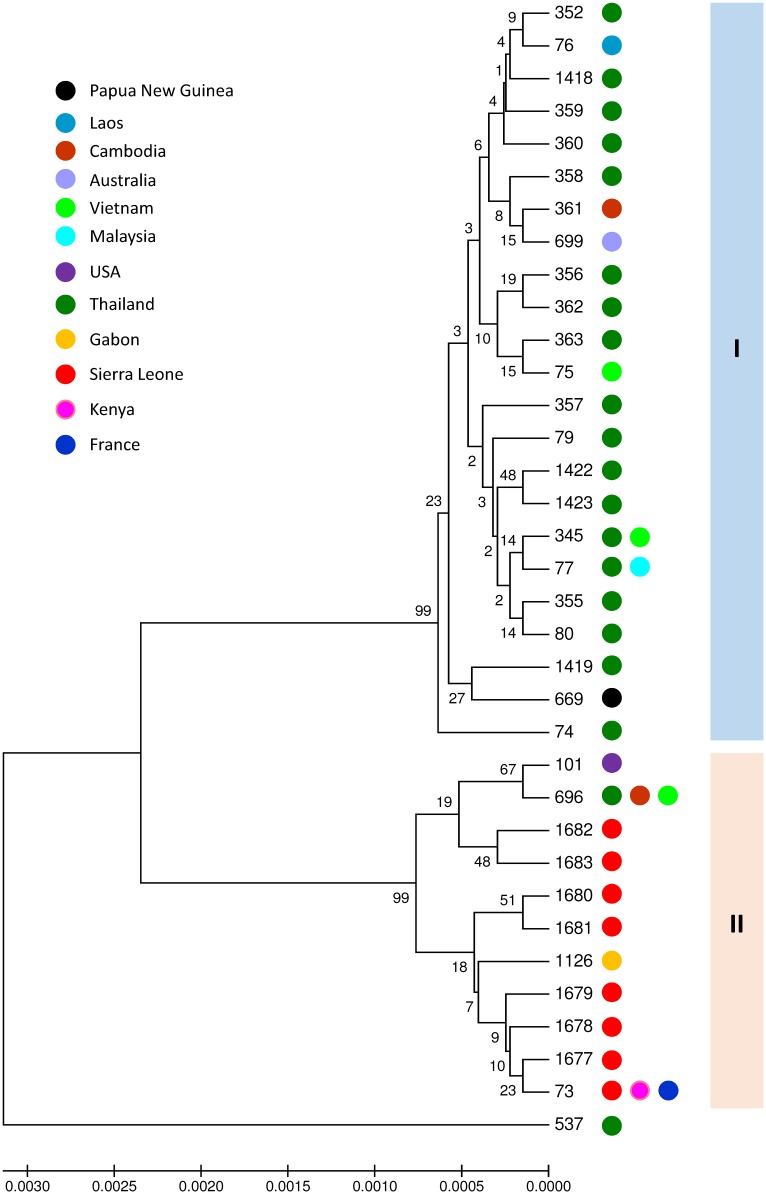
Phylogenetic tree of all *B*. *thailandensis* isolates. Phylogenetic tree of all *B*. *thailandensis* isolates with a unique sequence type recorded in the *B*. *pseudomallei* MLST website (https://pubmlst.org/bpseudomallei/). Phylogenetic tree is constructed based on the concatenated sequences of the seven household genes used for MLST using the UPGMA algorithm with the Jukes-Cantor model.

Two main clusters are presented in a phylogenetic tree based on the concatenated sequences of the seven household genes of all *B*. *thailandensis* STs available in the PubMLST database (Fig 2). Cluster I contains exclusively isolates from Asia and Oceania, while cluster II comprises isolates from all isolates from Sierra Leone and the one from Gabon (ST1126). One isolate with ST537 was an outlier. Interestingly, ST1126, ST696 and ST101 were identified to express a *B*. *pseudomallei*-like capsular polysaccharide (BTCV) [18] possibly explaining why many of the isolated *B*. *thailandensis* showed cross-reactivity with the *B*. *pseudomallei* latex-agglutination test. MLST data and microarray based comparative genomic hybridization revealed earlier that there is a separate subgroup of *B*. *thailandensis* isolates (ST696, ST101 and ST73) containing BTCV strains, which are genetically different from the other *B*. *thailandensis* isolates [17].

In this study, two clearly separated *B*. *thailandensis* clusters (I and II) were observed. Various studies have reported human infections by *B*. *thailandensis* belonging to both cluster I (ST77, ST80 and ST345) and cluster II (ST73 and ST101) (https://pubmlst.org/bpseudomallei/) [5–10]. It has been postulated that *B*. *thailandensis* isolates within cluster II are more virulent than those in cluster I [17], but evidence has not been reported [6,17]. Clinical characteristics are indistinguishable from *B*. *pseudomallei* infection and include soft tissue infection, abscess formation, pneumonia and sepsis [5–10]. The most recently described *B*. *thailandensis* case occurred in a 29-year old diabetic woman with an infected wound and swelling of her forearm after a car incident in Arkansas, US [6].

Our study has several limitations, including the lack of standard blood culture services for febrile patients across Sierra Leone. This limits targeted soil sampling studies centred around an index case. In addition, we cannot dismiss the possibility of sampling-error, although consensus guidelines were followed [14]. More knowledge about the exact composition of soils in which *B*. *pseudomallei* and *B*. *thailandensis* reside could help to determine which sites to study in future environmental surveillance studies. Furthermore, bacteria could have been present in a viable, but not cultivable state. It remains difficult to differentiate *B*. *thailandensis* and other members of the *B*. *pseudomallei* complex from *B*. *pseudomallei* by methods commonly available in clinical labs, even in developed countries, which may result in diagnostic confusion. Therefore, improving the detection and differentiation of members of the *B*. *pseudomallei* complex to improve patient care and appropriate public health responses is desired. Taken together, *B*. *pseudomallei* was not cultured from the soil of central Sierra Leone, but *B*. *thailandensis* with novel genotypes were found. *B*. *thailandensis* infection in humans have been sporadically reported in the literature in both the US and Asia [5–10]. *B*. *thailandensis* in general is considered a-virulent. As a result, clinical disease attributed to *B*. *thailandensis* is important. This also holds true for to the melioidosis research community, because no strict biocontainment conditions for *B*. *thailandensis* are required. The true clinical relevance of this soil-dwelling bacillus, however, remains to be elucidated. We encourage further environmental and sequencing studies on both *B*. *pseudomallei* and *B*. *thailandensis* to further understand the genetic diversity, virulence and evolution of these emerging organisms.

## Supporting information

S1 Table MLST analysis different suspected *Burkholderia* strains. (DOCX)

S1 Fig Schematic overview of the culture detection method used in this study. (DOCX)

S1 Supporting Methods DNA extraction Burkholderia strains. (DOCX)
