# Supplementary material for: Identification of Burkholderia thailandensis with novel genotypes in the soil of central Sierra Leone
Source: PLoS Negl Trop Dis. 2019 Jun 14;13(6):e0007402. doi: 10.1371/journal.pntd.0007402 (PMC6623504; doi:10.1371/journal.pntd.0007402)
Supplement: S1 Table — (DOCX) [file pntd.0007402.s001.docx]

**Table S1. MLST analysis different suspected *Burkholderia* strains.** MLST data analysis was performed based on partial sequences of seven housekeeping genes (see abbreviations). In total eight different sequencing types of *B. thailandensis* were found in the soil of Sierra Leone. The different colours indicate different sequencing types of *B. thailandensis*. Abbreviations: *ace* = acetoacetyl coenzyme A reductase; *gltB =* glutamate synthase; *gmhD =* ADP-L-glycero-D-manno-heptose 6-epimerase; *lepA =* GTP-binding elongation factor; *lipA =* lipoic acid synthetase; *narK* = nitrite extrusion protein; *ndh =* NADH dehydrogenase; *ST:* sequence type.

| **Isolate number** | ***ace*** | ***gltB*** | ***gmhD*** | ***lepA*** | ***lipA*** | ***narK*** | ***ndh*** | **ST** | **locus variant of ST73** | **Isolated from site** |
| --- | --- | --- | --- | --- | --- | --- | --- | --- | --- | --- |
|  |  |  |  |  |  |  |  |  |  |  |
|  |  |  |  |  |  |  |  |  |  |  |
| B78a | 5 | 5 | 9 | 5 | 7 | 7 | 5 | 73 | - | B |
| B78b | 5 | 5 | 9 | 5 | 7 | 7 | 5 | 73 | - | B |
| B32c | 5 | 5 | 9 | 5 | 7 | 7 | 5 | 73 | - | B |
| B32a | 5 | 5 | 9 | 5 | 7 | 7 | 5 | 73 | - | B |
| A34 | 5 | 5 | 9 | 5 | 7 | 138 | 5 | 1680 | SLV | A |
| A14a | 5 | 5 | 9 | 5 | 7 | 138 | 5 | 1680 | SLV | A |
| A98 | 5 | 5 | 9 | 5 | 7 | 138 | 5 | 1680 | SLV | A |
| A15a | 5 | 5 | 9 | 5 | 7 | 138 | 5 | 1680 | SLV | A |
| A36a | 5 | 5 | 9 | 123 | 7 | 138 | 5 | 1681 | DLV | A |
| A15b | 5 | 5 | 9 | 5 | 7 | 7 | 14 | 1677 | SLV | A |
| B32b | 5 | 5 | 9 | 5 | 7 | 7 | 14 | 1677 | SLV | B |
| A39a | 5 | 5 | 9 | 5 | 7 | 7 | 14 | 1677 | SLV | A |
| A37a | 5 | 5 | 9 | 5 | 7 | 7 | 14 | 1677 | SLV | A |
| A35 | 5 | 5 | 9 | 5 | 7 | 7 | 14 | 1677 | SLV | A |
| A9 | 5 | 5 | 9 | 5 | 7 | 7 | 14 | 1677 | SLV | A |
| A41 | 5 | 5 | 9 | 5 | 7 | 7 | 14 | 1677 | SLV | A |
| A14b | 5 | 5 | 9 | 5 | 7 | 7 | 14 | 1677 | SLV | A |
| A65 | 5 | 5 | 9 | 5 | 7 | 7 | 14 | 1677 | SLV | A |
| A18b | 5 | 5 | 9 | 5 | 7 | 7 | 14 | 1677 | SLV | A |
| B78c | 5 | 5 | 9 | 5 | 7 | 7 | 14 | 1677 | SLV | B |
| A10a | 5 | 5 | 9 | 5 | 7 | 7 | 14 | 1677 | SLV | A |
| A97b | 103 | 5 | 9 | 5 | 7 | 7 | 14 | 1678 | DLV | A |
| A62 | 103 | 5 | 9 | 5 | 7 | 7 | 14 | 1678 | DLV | A |
| A56a | 5 | 5 | 9 | 5 | 7 | 20 | 14 | 1679 | DLV | A |
| B67a | 5 | 5 | 9 | 5 | 7 | 20 | 14 | 1679 | DLV | B |
| B67b | 5 | 5 | 9 | 5 | 7 | 20 | 14 | 1679 | DLV | B |
| B67c | 5 | 5 | 9 | 5 | 7 | 20 | 14 | 1679 | DLV | B |
| A1 | 6 | 5 | 9 | 5 | 7 | 137 | 14 | 1682 | DLV | A |
| A2 | 6 | 5 | 9 | 5 | 7 | 137 | 14 | 1682 | DLV | A |
| A5 | 6 | 5 | 9 | 5 | 7 | 137 | 14 | 1682 | DLV | A |
| A3 | 6 | 5 | 9 | 5 | 7 | 137 | 14 | 1682 | DLV | A |
| A4 | 6 | 5 | 9 | 5 | 183 | 136 | 14 | 1683 | TLV | A |
