# Supplementary material for: Identification of Burkholderia thailandensis with novel genotypes in the soil of central Sierra Leone
Source: PLoS Negl Trop Dis. 2019 Jun 14;13(6):e0007402. doi: 10.1371/journal.pntd.0007402 (PMC6623504; doi:10.1371/journal.pntd.0007402)
Supplement: S1 Fig — (DOCX) [file pntd.0007402.s002.docx]

**Supporting information methods**

## DNA extraction *Burkholderia* strains

Genomic (g)DNA of heat-killed *Burkholderia* was extracted using the Maxwell® 16 Blood DNA purification kit (Promega, Leiden, The Netherlands) according manufacturer’s instructions. In short: STAR buffer (a lysis buffer) was added to the heat killed bacteria and homogenized at 5.5 millisecond (ms) or 3x 1 minute using the Precellys ®24 homogenizer (Bertin Instruments, Montigny-le-Bretonneux, France). Subsequently, the samples were heated for 15 minutes at 95 °C at 1000 rpm, centrifuged for 5 minutes at 14000 rpm at 4 °C and supernatant was collected. These steps were repeated once. DNA extraction from the supernatant was performed with the Maxwell® 16 instrument (Promega, Leiden, The Netherlands) using the Maxwell RSC blood DNA mode. Extracted DNA was diluted in 60 µL nuclease-free water. The amount of DNA extracted per strain (ng/µL) was determined using the NanoDrop^TM^ 2000/2000c Spectophotometer (Thermo Fisher Scientific, Waltham, USA) at 260/280 or Qubit® 2.0 fluorometer (Thermo Fisher Scientific, Waltham, USA). DNA extractions were stored at -20 °C.
