# Supplementary material for: Identification of Burkholderia thailandensis with novel genotypes in the soil of central Sierra Leone
Source: PLoS Negl Trop Dis. 2019 Jun 14;13(6):e0007402. doi: 10.1371/journal.pntd.0007402 (PMC6623504; doi:10.1371/journal.pntd.0007402)
Supplement: S1 Supporting Methods — (DOCX) [file pntd.0007402.s003.docx]

**Supporting information methods**

## DNA extraction *Burkholderia* strains

Genomic (g)DNA of heat-killed *Burkholderia* was extracted using the Maxwell® 16 Blood DNA purification kit (Promega, Leiden, The Netherlands) according manufacturer’s instructions. In short: STAR buffer (a lysis buffer) was added to the heat killed bacteria and homogenized at 5.5 millisecond (ms) or 3x 1 minute using the Precellys ®24 homogenizer (Bertin Instruments, Montigny-le-Bretonneux, France). Subsequently, the samples were heated for 15 minutes at 95 °C at 1000 rpm, centrifuged for 5 minutes at 14000 rpm at 4 °C and supernatant was collected. These steps were repeated once. DNA extraction from the supernatant was performed with the Maxwell® 16 instrument (Promega, Leiden, The Netherlands) using the Maxwell RSC blood DNA mode. Extracted DNA was diluted in 60 µL nuclease-free water. The amount of DNA extracted per strain (ng/µL) was determined using the NanoDrop^TM^ 2000/2000c Spectophotometer (Thermo Fisher Scientific, Waltham, USA) at 260/280 or Qubit® 2.0 fluorometer (Thermo Fisher Scientific, Waltham, USA). DNA extractions were stored at -20 °C.

## DNA extraction soil samples

Soil samples containing *B. thailandensis* and suspected *B. pseudomallei* were selected for DNA extraction (site A: 27 samples, site B: 3 samples). In addition, twenty-two samples of site B and ten samples of each of the other sampling sites (site C t/m J) were selected using random sampling methods. Four soil samples from the Amsterdam area were used as negative control. The DNA extraction was performed with Maxwell®FSC DNA IQ^TM^ Casework Kit: Low Elution Volume (LEV) mode (Promega, Leiden, the Netherlands), with some small modifications. In brief: STAR buffer was added to the soil samples and mixture was homogenized at 5.5 ms for 3x one minute using the Precellys ®24 homogenizer (Bertin Instruments, Montigny-le-Bretonneux, France). Subsequently, the samples were centrifuged for five minutes at 14,000 rpm at 4 °C and the supernatant was collected. To prevent degradation and absorption of nucleic acids during DNA extraction, 5% dry milk solution was added to the supernatant. DNA extraction from the supernatant was performed with the Maxwell® 16 instrument (Promega, Leiden, the Netherlands) using the FSC DNA IQ^TM^ Casework Kit custom mode v.0.9.1. Extracted DNA was diluted in 60 µL elution buffer. The amount (ng/ µL) of extracted DNA per sample was determined using NanoDrop^TM^ 2000/2000c Spectrophotometer (Thermo Fisher, Waltham, USA) at 260/280 nm. Samples were stored at -20°C.

## qPCR for detection of *B. pseudomallei*

Molecular detection of *B. pseudomallei* was assessed by a qPCR targeting *TTSS1* and a duplex qPCR targeting *BPSS0087* and *BPSS0745* as described elsewhere [1] with slight adjustments. The qPCRs contained target specific fluorescent probes. The TTSS1 qPCR mixture, with a final reaction volume of 12 µL, consisted of Applied Biosystem^TM^ 2X TaqMan^TM^ Universal PCR Master Mix (Thermo Fisher, Waltham, USA) diluted to 1X, 400 nM of the forward and reverse primer, 260 nM of the fluorescent probe, 20 µg/µL BSA dilution and 3 µL of the sample DNA. Cycling conditions were: one cycle of 95°C for ten minutes; 60 cycles of 95°C for 15 seconds, 60°C for 30 seconds. The *BPSS0087-BPSS0745*-qPCR mixture, with a total reaction volume of 12 µL, consisted of Applied Biosystem^TM^ 2X TaqMan^TM^ Universal PCR Master Mix (Thermo Fisher, Waltham, USA) diluted to 1X, 500 nM of the forward primers, 400nM of the reverse primers, 180 nM of the *BPSS0087* fluorescent probe, 200 nM of the *BPSS0745* fluorescent probe, 20 µg/µL BSA dilution and 3 µL of the sample DNA. Cycling conditions were: one cycle of 95°C for 10 minutes; 65 cycles of 95°C for 15 seconds, 57°C for 30 seconds, 60°C for 30 seconds. Both qPCRs were performed using the Light Cycler 480 instrument II (Roche) and analyzed with help of the Light Cycler® 480 Software release 1.5.1.62 SP3.

## Quantification of bacterial load and *B. pseudomallei*

A standard curve of the gDNA of *B. pseudomallei* K96243, serial diluted over six orders of magnitude, was used in each *B. pseudomallei* specific qPCR to calculate the amount of *B. pseudomallei* in the specific samples. Because the qPCRs each target single copy regions of the *B. pseudomallei* genome, the number of copies determined by qPCR can be converted into genome equivalents (GE) as described elsewhere [1].


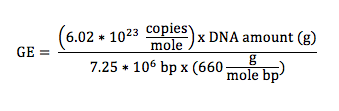


All qPCRs reactions were analyzed in duplex and standard curves were used in triplicate and mean valuated to obtain a valid standard curve. The mean of the duplex qPCR reactions represented the *B. pseudomallei* load in the soil samples. A schematic overview of this molecular detection method is shown in Fig S1.

**References**

1. Gohler A, Trung TT, Hopf V, et al. Multitarget Quantitative PCR Improves Detection and Predicts Cultivability of the Pathogen *Burkholderia pseudomallei.* Appl Environ Microbiol. 2017;83.
